# Quantification of hepatocellular carcinoma heterogeneity with multiparametric magnetic resonance imaging

**DOI:** 10.1038/s41598-017-02706-z

**Published:** 2017-05-26

**Authors:** Stefanie J. Hectors, Mathilde Wagner, Octavia Bane, Cecilia Besa, Sara Lewis, Romain Remark, Nelson Chen, M. Isabel Fiel, Hongfa Zhu, Sacha Gnjatic, Miriam Merad, Yujin Hoshida, Bachir Taouli

**Affiliations:** 1 Translational and Molecular Imaging Institute, Icahn School of Medicine at Mount Sinai, New York, NY United States; 2 Department of Radiology, Icahn School of Medicine at Mount Sinai, New York, NY United States; 3 Sorbonne Universités, UPMC, Department of Radiology, Hôpital Pitié-Salpêtrière, Assistance Publique-Hôpitaux de Paris, Paris, France; 4 Immunology Institute, Icahn School of Medicine at Mount Sinai, New York, NY United States; 5 Department of Pathology, Icahn School of Medicine at Mount Sinai, New York, NY United States; 6 Oncological Science, Icahn School of Medicine at Mount Sinai, New York, NY United States; 7 Department of Medicine/Division of Liver Diseases, Icahn School of Medicine at Mount Sinai, New York, NY United States

## Abstract

Tumour heterogeneity poses a significant challenge for treatment stratification. The goals of this study were to quantify heterogeneity in hepatocellular carcinoma (HCC) using multiparametric magnetic resonance imaging (mpMRI), and to report preliminary data correlating quantitative MRI parameters with advanced histopathology and gene expression in a patient subset. Thirty-two HCC patients with 39 HCC lesions underwent mpMRI including diffusion-weighted imaging (DWI), blood-oxygenation-level-dependent (BOLD), tissue-oxygenation-level-dependent (TOLD) and dynamic contrast-enhanced (DCE)-MRI. Histogram characteristics [central tendency (mean, median) and heterogeneity (standard deviation, kurtosis, skewness) MRI parameters] in HCC and liver parenchyma were compared using Wilcoxon signed-rank tests. Histogram data was correlated between MRI methods in all patients and with histopathology and gene expression in 14 patients. HCCs exhibited significantly higher intra-tissue heterogeneity vs. liver with all MRI methods (*P* < 0.030). Although central tendency parameters showed significant correlations between MRI methods and with each of histopathology and gene expression, heterogeneity parameters exhibited additional complementary correlations between BOLD and DCE-MRI and with histopathologic hypoxia marker HIF1α and gene expression of Wnt target *GLUL*, pharmacological target *FGFR4*, stemness markers *EPCAM* and *KRT19* and immune checkpoint *PDCD1*. Histogram analysis combining central tendency and heterogeneity mpMRI features is promising for non-invasive HCC characterization on the imaging, histologic and genomics levels.

## Introduction

Quantitative functional multiparametric magnetic resonance imaging (mpMRI) of cancer allows for non-invasive assessment of several tumour characteristics, such as cellularity, perfusion and oxygenation, which can be used for tumour characterization and for assessing treatment response^1^. Apparent diffusion coefficient (ADC) quantification using diffusion-weighted imaging (DWI) has shown to correlate with tumour cellularity^2^. Tissue oxygenation can be indirectly assessed using blood oxygenation level-dependent (BOLD) and tissue oxygenation level-dependent (TOLD) MRI^3^. Tissue perfusion and flow can be quantitatively measured using dynamic contrast-enhanced MRI (DCE-MRI)^4^.

Many studies that employ mpMRI to assess/predict tumour response use central tendency parameters, such as mean or median, over entire regions of interest (ROIs) to determine longitudinal changes in the tumour tissue after treatment^5^. However, such analysis may not represent the exact tumour status, given the intrinsic heterogeneous tumour composition^5^. Heterogeneity analysis of tumour MRI measurements may provide accurate markers of tumour heterogeneity at the genetic, cellular and molecular levels^5^ and thereby allow for a better understanding of tumour characteristics that may affect treatment decisions.

Hepatocellular carcinoma (HCC) lesions are known to exhibit substantial intra- and inter-tumour heterogeneity, due to a large variety in etiological and genetic backgrounds and the long-time development of the disease^6^. Tumour heterogeneity poses a significant challenge for treatment stratification in HCC. While pathological and genetic heterogeneity in HCC lesions have been described^7^, imaging reports on HCC heterogeneity are extremely limited, with only one study reporting visual assessment of HCC heterogeneity on contrast-enhanced MRI^8^, with no study employing quantitative imaging measurements of HCC heterogeneity.

Tumour imaging phenotypes, including histogram features, may potentially correlate with the underlying genotype. Thus, non-invasive imaging, including computed tomography (CT) and MRI, can potentially be used as a surrogate for genomics and transcriptomics (radiogenomics)^9–11^. Radiogenomics could potentially be used for the prediction of certain molecular gene signatures^12^. A major advantage of imaging versus genomics or histopathological analysis is that imaging captures the entire tumour non-invasively^12^. In addition, combined analysis of imaging, histopathology and genomics data may improve characterization of tumours, because next to mutual information, the separate assessments may also provide additional, independent information on tumour properties, which may help predict outcomes^12^.

Immunotherapy has recently revolutionized treatment in cancer^13, 14^. The clinical experience with immunotherapy in HCC is growing, with recent and ongoing phase I trials^15, 16^. The success of such treatment heavily depends on tumour expression of immunotherapy targets, such as immune checkpoints. Identification of imaging features that correlate with gene expression of immunotherapy targets potentially allows for non-invasive prediction of immunotherapy outcome.

The objectives of our study were to: 1) quantify intra- and inter-tumour heterogeneity of HCC lesions using mpMRI, consisting of DCE-MRI, BOLD-MRI, TOLD-MRI and DWI, and 2) report our preliminary results correlating the quantitative MRI parameters with advanced histopathology and gene signatures and expression levels in a subset of patients.

## Patients and Methods

### Patients

This single-centre prospective study was compliant with the Health Insurance Portability and Accountability Act and approved by the institutional review board at the Icahn School of Medicine at Mount Sinai. Written informed consent was obtained from all subjects. From June 2013 to June 2016, 41 consecutive patients with HCC were enrolled. HCC was diagnosed based on routine imaging by two radiologists in consensus [observer 1 (MW) and observer 2 (CB), with 5 and 6 years of experience in body MRI, respectively], according to the Organ Procurement and Transplantation Network (OPTN) criteria^17^. Of the 41 initial patients, 9 patients were excluded because of severe motion in the DCE-MRI acquisition (n = 5), absence of contrast injection (n = 2) or low quality of BOLD-MRI data (n = 2). Thus, 32 patients were finally included (M/F 26/6, mean age 59 y, range 30–71 y), of which 31 had chronic liver disease [chronic hepatitis C (n = 18), chronic hepatitis B (n = 9), non alcoholic steatohepatitis (n = 2), cryptogenic cirrhosis (n = 1) and alcoholic cirrhosis (n = 1)]. For one patient without history of liver disease, HCC was confirmed at histopathology. Three patients underwent locoregional therapy with either transarterial chemoembolization (TACE) combined with radiofrequency ablation (n = 1), TACE only (n = 1) or radioembolization (n = 1) before MRI (range 111–178 days). Data of 23 of the included patients has been published in at least one of our previous publications^3, 18, 19^. The aim of the previous studies was mainly to quantify each of the individual MRI methods in HCC lesions and to assess test-retest repeatability. Assessment of tumour heterogeneity was beyond the scope of those studies.

### MRI acquisition

The MRI acquisition was performed on 1.5 T (Siemens Aera, Siemens Healthineers, Erlangen, Germany; n = 19) or 3.0 T [Siemens Skyra (n = 5) or Siemens BioGraph mMR (n = 8)] systems. Each of the systems is equipped with a 32-channel spine and flexible body array coil for signal reception. Subjects were asked to fast for 6 hours to eliminate post-prandial effects on portal venous blood flow^20^. In addition to axial DWI, BOLD-MRI, TOLD-MRI and DCE-MRI, the MRI acquisition consisted of axial and coronal T_2_-weighted imaging, axial dual-echo chemical shift imaging, T_1_-weighted imaging before and at a delayed phase after contrast injection. The acquisition parameters for DWI, BOLD-MRI, TOLD-MRI and DCE-MRI are listed in Table 1. For BOLD-MRI and TOLD-MRI, R_2_* and R_1_ acquisitions were performed before and at the end of a respiratory oxygen challenge of 10–15 minutes at a flow rate of 15 l/min^3^. DCE-MRI acquisition consisted of 100 dynamic acquisitions. Eight seconds after the start of the acquisition, a half dose (0.05 mmol/kg) of gadobenate dimeglumine (Multihance, Bracco Diagnostics Inc) followed by a 25 ml saline flush was administered intravenously at a rate of 3 ml/s^19^. Half dose of the contrast agent was used to avoid saturation of the signal intensity in the DCE-MRI acquisition, because of the high T_1_ relaxivity of the contrast agent.

**Table 1:** Acquisition parameters of the multiparametric MRI protocol.

	DWI	BOLD				TOLD			DCE-MRI
Sequence type	Single-shot spin-echo EPI	2D MGRE	2D MGRE	2D MGRE	2D MGRE	3D VFA	2D IR-LL	2D-IR-LL	3D FLASH
Acquisition plane	Axial	Axial	Axial	Axial	Axial	Axial	Axial	Axial	Axial
MR system	All	Skyra (n = 5)	Aera (n = 10)	Aera (n = 9)	mMR (n = 8)	Skyra (n = 5)	Aera (n = 19)	mMR (n = 8)	All
TE (ms)	74–81	2.5, 4.9, 7.4, 9.8, 12.3, 14.8, 17.2	4.8, 9.5, 14.3, 19.1, 23.9	1.7, 2.9, 4.3, 5.6, 6.9, 10.0, 15.0, 20.0, 25.0, 30.0, 35.0, 40.0	1.1, 2.4, 3.8, 5.2, 6.6, 8.0, 10.0, 12.0, 15.0, 20.0, 25.0, 30.0	1.2	1.0	1.2	1.0
TR (ms)	4500 (Skyra); one respiration (Aera/mMR);	165	242	319	249	10	2.3	35.1	2.7
FA (°)	90	35	35	20	18	1, 10, 19	8	10	9.5–11.5
TI (ms)	—	—	—	—	—	—	42–1577 (32 TI’s)	80–1445 (40 TI’s)	—
b-values (s/mm^2^)	0, 15, 30, 45, 60, 75, 90, 105, 120, 135, 150, 175, 200, 400, 600, 800	—	—	—	—	—	—	—	—
FOV (mm^2^)	380 × 255	340 × 255	340 × 255	340 × 255	340 × 255	350 × 260	420 × 290	380 × 280	380 × 280
Matrix	320 × 240	512 × 384	384 × 288	384 × 288	384 × 288	384 × 288	128 × 88	384 × 288	384 × 288
Slice thickness (mm)	7–8	7	7	7	7	5	8	8	4–5
Number of slices	20–30	4–5	4–5	4–5	4–5	36	1–2	1	40
Acceleration factor	2	2	2	2	2	2	—	—	4
Acquisition time (min:s)	8:00	0:15	0:15	0:15	0:15	0:14	0:18	0:10	0:02 per dynamic

EPI = echo planar imaging, FA = flip angle, FLASH = fast low angle shot, FOV = field-of-view, IR-LL = inversion recovery Look-Locker, MGRE = multi-echo gradient echo, TE = echo time, TI = inversion time, TR = repetition time, VFA = variable flip angle.

### Image analysis

Image analysis was performed on MATLAB platform (version R2015a, MathWorks, Natick, MA, USA), unless specified otherwise. The analysis was performed by two observers in consensus [observer 1 and observer 3 (SH), an MRI physicist with 6 years of experience].

#### DWI analysis

DWI acquisition was performed with 16 b-values for the purpose of the estimation of intravoxel incoherent motion (IVIM) diffusion parameters^21, 22^. However, for this particular study, we only used 3 b-values (60, 400 and 800 s/mm^2^) to determine the apparent diffusion coefficient (ADC), which was estimated as the slope of a linear fit of the logarithmic signal intensity (SI) curves at these particular b-values.

#### BOLD/TOLD analysis

R_2_* maps were generated by fitting of a mono-exponential decay model to the pixel signal values at all acquired echoes^3^. T_1_ maps were calculated using the Look-Locker recovery equation^23^ or the Ernst equation for a spoiled gradient recalled echo (SPGR) sequence for the variable flip angle measurements^24^. The pixel T_1_ values were inverted to obtain R_1_ values. In addition, ΔR_1_ and ΔR_2_* maps were generated by subtraction of the baseline R_1_ and R_2_* measurements from the measurements after oxygen (ΔR_1/2*_ = R_1/2*_ post O_2_ − R_1/2*_ pre O_2_)^3^.

#### DCE-MRI analysis

Prior to pharmacokinetic analysis, motion correction was applied on the DCE-MR images with 3D rigid registration using open-source image analysis software (Firevoxel, CAI^2^R, New York University, New York, NY, USA). Free-hand 3D regions-of-interest (ROIs) were drawn in the portal vein on the registered images and in the abdominal aorta at the level of the celiac trunk on the unregistered images. Pixel dynamic SI curves were converted to dynamic contrast agent concentration ([CA]) curves by using the signal equation for a SPGR sequence, pre-contrast R_1_ values as determined from the baseline TOLD measurements (registered to the DCE-MRI measurements, see below) and the contrast agent’s relaxivity^25^. For the portal vein and aorta, pre-contrast R_1_ values were based on literature^26^. A haematocrit value of 0.45 was used for conversion from blood [CA] to plasma [CA]. Contrast agent relaxivity values of 8.1 mM^−1^ s^−1^ and 6.3 mM^−1^ s^−1^ were used at 1.5 T and 3.0 T, respectively^27^.

Pharmacokinetic parameter maps were generated by fitting of the dual-input signal compartment model to the dynamic [CA] curves in each pixel^28^. The following parameters were estimated in tumours and liver parenchyma: arterial flow (F_a_), portal flow (F_p_), total flow (F_t_ = F_a_ + F_p_), arterial fraction (ART = F_a_/F_t_), mean transit time (MTT) and the distribution volume fraction of the contrast agent through the tissue (DV).

#### Coregistration of mpMRI data

Although the orientation of all sequences was in the axial plane, there were acquisition differences related to different matrix size, field of view, offset and subject motion. Therefore, a coregistration algorithm (MATLAB function fitgeotrans) was used to register the mpMRI data. Landmark points were placed on both the raw DCE-MR images and the corresponding BOLD, TOLD or ADC parameter maps. Subsequently, nonrigid coregistration was performed, visually checked and repeated if necessary. The coregistration was only performed in slices that contained HCC lesion(s) and that were common to all acquisitions.

#### Region of interest (ROI) analysis

ROIs in the HCC lesions and liver parenchyma were placed by observer 1 on the registered DCE-MR images. For the HCC ROIs, the entire lesion was included, both viable and, if present, necrotic regions. Subsequently, the ROIs were copied and pasted on the BOLD, TOLD and DWI parameter maps and adjusted if necessary to avoid inclusion of artefacts. Histogram quantification included determination of central tendency (mean and median) and heterogeneity [standard deviation (SD), kurtosis and skewness] parameters. Kurtosis determines the peakedness of the distribution. A higher kurtosis has been associated with increased heterogeneity^29^, because distributions with high peaks often have fat tails of outlier values. A skewness value deviating from zero indicates that there is tail toward either the left or right side of the distribution, which is also indicative of heterogeneity.

### Histopathology

**Figure 1:**
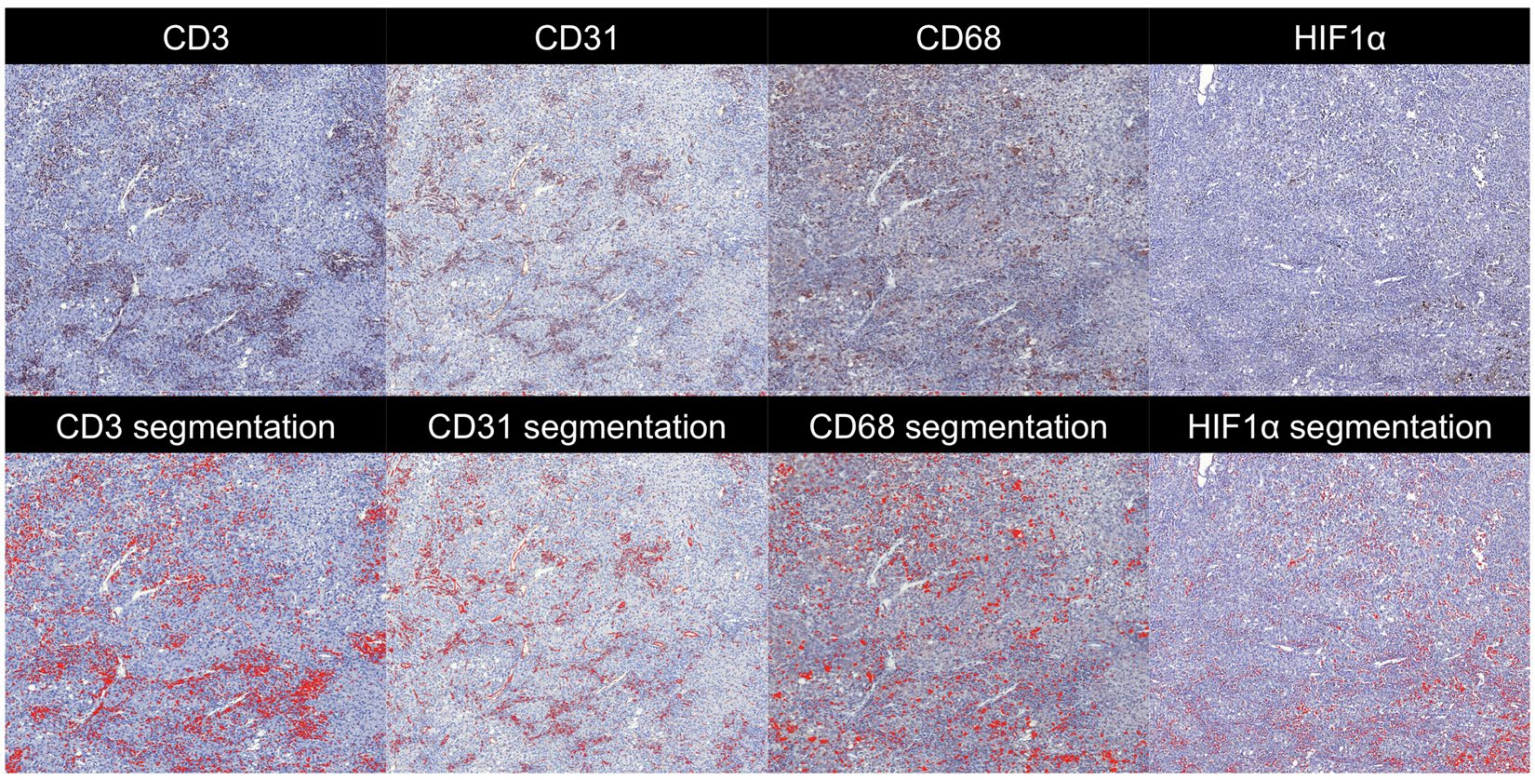
Histopathology processing of HCC sections obtained from a 70-year-old male patient with NASH cirrhosis and HCC. Zoomed microscopy images (×20) of an HCC section stained using Multiplexed Immunohistochemical Consecutive Staining on Single Slide (MICSSS) for CD3, CD31 and CD68 and a separate section stained for HIF1α are shown in the top row. Results of the automatic segmentation algorithm are shown in the bottom row, in which pixels identified as stained are coloured in red. CD3 = cluster of differentiation 3, CD31 = cluster of differentiation 31, CD68 = cluster of differentiation 68, HIF1α = hypoxia-inducible factor 1-alpha.

Fifteen patients underwent partial hepatectomy after the MRI examination (time range between MRI and resection 0–40 days) and two patients had a biopsy of their index HCC lesion (57 days before and 15 days after the MRI exam). Histological grade^30^ was available for the HCC lesions in these 17 patients. For one patient that underwent resection, no sections were available since the resection was performed in an outside hospital. For each HCC lesion in the 14 remaining resected liver samples, two five-μm-thick formalin-fixed paraffin-embedded sections were available for advanced histopathological staining. The Multiplexed Immunohistochemical Consecutive Staining on Single Slide (MICSSS)^31^ technique was used to sequentially stain endothelial cells (CD31 monoclonal antibody, clone JC70), macrophages (CD68, clone KP1) and T-cells (CD3, clone 2GV6). For detection of hypoxia, a separate HCC section stained for hypoxia-inducible factor 1-alpha (HIF1α) activity was used for each lesion^32^. The stained HCC sections were digitally imaged at 20x magnification using a whole-slide scanner (Olympus, Tokyo, Japan). A threshold-based segmentation method was implemented in MATLAB to detect stained pixels **(**Fig. 1 **)**. For the segmentation, the red, green and blue intensity values from the images were retrieved and scaled from 0 to 255. Since the stained pixels generally had a dark red appearance, a pixel was considered stained if (1) the red intensity was higher than 1.1 times the green channel and (2) the average signal intensity was lower than 120. These thresholds were empirically chosen based on inspection of all histological images. From these segmentations, stained tumour fractions were derived.

### Gene expression analysis

Histologically distinct components of the 14 resected HCC tumours were macro-dissected using H&E staining of serial tissue sections as reference as previously described^33^. Isolated total RNA samples were profiled to determine transcriptomic HCC subtypes S1, S2 and S3^34^ with digital transcript counting technology using the nearest template prediction algorithm as previously described^33^. In addition, the following HCC marker genes were simultaneously profiled: liver specific Wnt target overexpressed in S3 subtype (*GLUL*)^35^, stemness markers (*EPCAM, KRT19*)^36, 37^, early HCC markers (*BIRC5, HSP70, LYVE1, EZH2*)^38, 39^, a pharmacological target currently under clinical testing (*FGFR4*)^40^, potentially targetable angiogenesis marker (*VEGFA*)^41^ and targetable immune checkpoints (*CFfigD274, PDCD1, CTLA4*)^42^.

### Statistical analysis

All values are presented as mean ± SD. For all correlation analyses, the Spearman correlation test was used. For all tests, a *P*-value lower than 0.05 was considered significant. For the analyses of differences and correlations between techniques, TOLD parameters, except ΔR_1_, were not compared with the DCE-MRI parameters, because the baseline R_1_ measurements were used as input for the DCE-MRI modelling.

#### Intra-tumour heterogeneity

Differences in kurtosis and skewness values between parameters of different MRI techniques were tested for significance using a Friedman test followed by post-hoc Bonferroni-corrected Wilcoxon signed rank tests. Mean, median and SD values were not compared between the techniques, because they directly depend on the actual MRI parameter values, which are inherently different between the MRI techniques. Liver and HCC histogram parameter values were compared using Wilcoxon signed-rank tests. For patients with multiple HCC lesions, the histogram features of the different lesions were averaged for comparison with liver parenchyma.

#### Inter-tumour heterogeneity

The absolute coefficient of variation (CV = 100*SD/absolute mean) between all MRI parameter features across lesions was measured. Absolute CV was calculated because of the presence of negative skewness values.

#### Correlation between MRI methods

The correlation of the mean, median, SD, kurtosis and skewness measures between the parameters of different MRI methods was assessed. The correlation analysis between mean and median parameters was performed both on all lesions as well as separately for lesions measured at 1.5 T and 3.0 T. To assess the effect of field strength on the correlations, differences between the correlations at both field strengths were tested for significance using a Fisher transformation test.

#### Correlation with histopathology and gene expression levels

The MRI histogram features in the tumour ROIs were correlated with histopathology stained tumour fractions and gene expression levels. In addition, differences in MRI parameters between HCC grades and molecular subclasses were assessed for statistical significance using Kruskal-Wallis tests.

## Results

Forty-four HCCs were identified in 32 patients. Five lesions were excluded because they were not covered by TOLD/BOLD measurements (n = 4) or were completely necrotic due to previous treatment (n = 1). Two other treated lesions were included since they exhibited limited necrosis (<30%). The final 39 included lesions had an average size of 4.4 ± 3.3 cm (range 1.5–13 cm). The number of analysed lesions per patient was as follows: 1 (n = 27, 84%), 2 (n = 4, 13%) or 4 (n = 1, 3%). The distribution of histological grade for the lesions with pathologic validation was as follows: well differentiated (n = 2), moderately differentiated (n = 9), poorly differentiated (n = 5) and undifferentiated (n = 1).

### Intra-tumour heterogeneity

**Figure 2:**
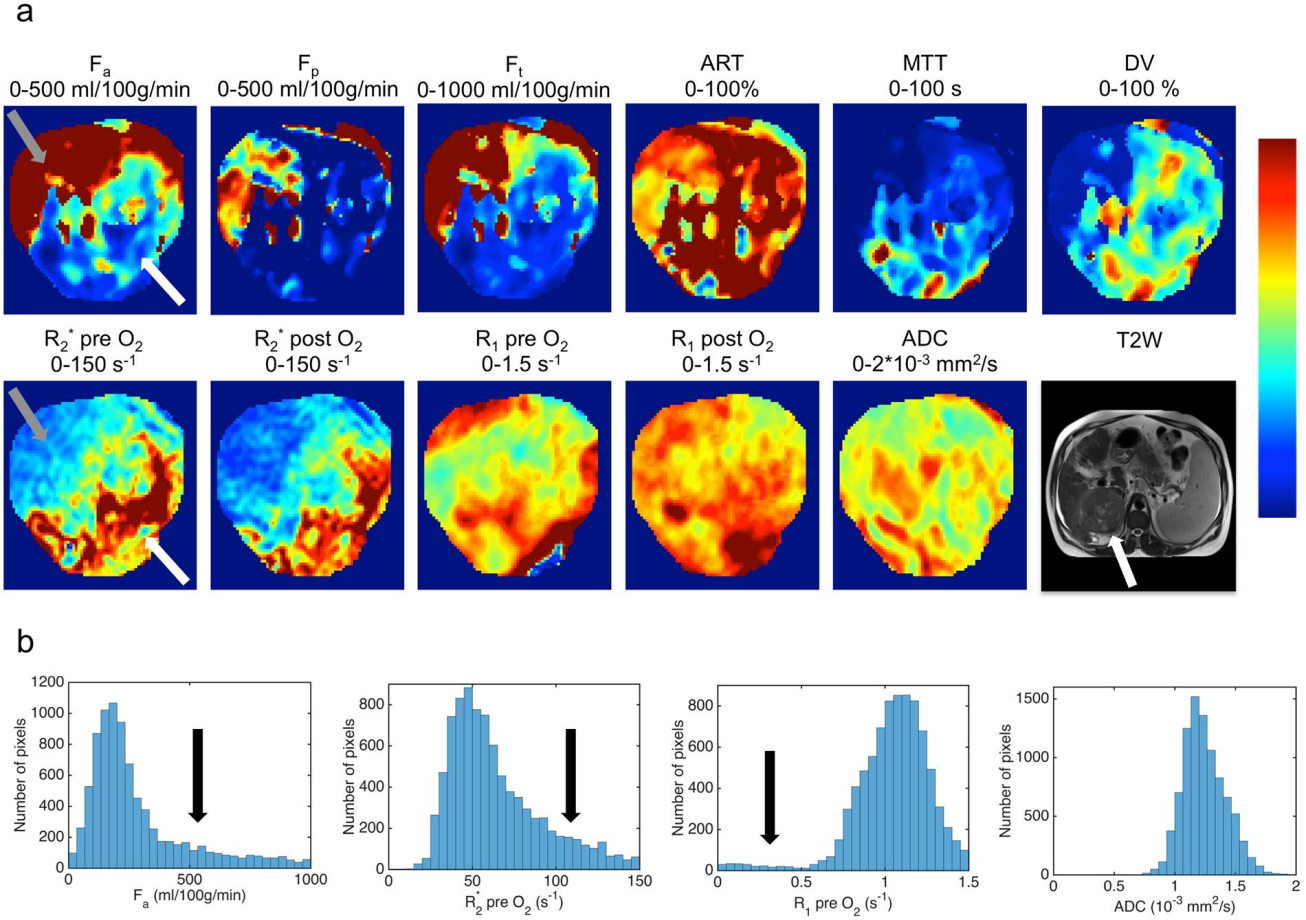
54 year-old male patient with cirrhosis secondary to chronic hepatitis B virus infection and HCC. (**a**) Representative magnified parametric maps of a large (8.3 cm) HCC. Location of the tumour within the liver is indicated by the white arrow on the T_2_-weighted image (bottom row, right). A distinct region in the anterior portion of the tumour of high arterial flow (F_a_) and low R_2_* was observed, reflective of high tumour perfusion and normoxia (grey arrow in F_a_ and R_2_* pre O_2_ maps). The posterior portion of the tumour displays low F_a_ and high R_2_*, suggestive of poor perfusion and hypoxia (white arrow in F_a_ and R_2_* pre O_2_ maps). (**b**) Histograms of F_a_, R_2_* pre O_2_, R_1_ pre O_2_ and ADC in the same lesion. The extensive heterogeneity observed in the parameter maps of F_a_ and R_2_* pre O_2_ is also reflected in the histograms, as illustrated by the fat tails and pronounced skewness, indicated by arrows. The R_1_ pre O_2_ histogram also exhibited skewness (black arrow). ADC = apparent diffusion coefficient, ART = arterial fraction, DV = distribution volume, F_a_ = arterial flow, F_p_ = portal flow, F_t_ = total flow, MTT = mean transit time, R_1_ = longitudinal relaxation rate, R_2_* = transverse relaxation rate.

Average mean, median, SD, kurtosis and skewness values for all assessed parameters in the liver parenchyma and HCC lesions are listed in Table 2. HCC lesions exhibited more intra-tissue heterogeneity compared to liver parenchyma (Fig. 2), with significantly higher SD (F_a_, F_p_, F_t_, MTT, DV and R_1_ pre O_2_; *P* < 0.020), kurtosis (F_p_ and ADC; *P* < 0.030) and skewness (F_p_, ART, R_2_* post O_2_ and R_1_ pre O_2_; *P* < 0.012). Kurtosis and skewness in HCC lesions were significantly different between MRI parameters of different techniques (*P* < 0.001). Specifically, significantly higher kurtosis was observed for F_p_, R_1_ post O_2_ and ΔR_1_ compared to ADC (*P* < 0.045). A significantly higher skewness was observed for F_p_ vs. ΔR_2_* (*P* = 0.020), while ART showed a significantly lower skewness compared to R_2_* post O_2_, ΔR_2_*, ΔR_1_ and ADC (*P* < 0.030). Leptokurtosis, i.e. a kurtosis value higher than 3, which is indicative of intra-tumour heterogeneity, was observed in all lesions for at least one MRI parameter. In 9 lesions (23%) leptokurtosis was seen in all MRI methods (DCE-MRI, BOLD, TOLD and DWI), while the remaining lesions showed leptokurtosis in a subset of MRI methods [DCE-MRI, BOLD and TOLD in 18 lesions (46%); DCE-MRI, TOLD and DWI in 4 lesions (10%); DCE-MRI and TOLD in 4 lesions (10%); BOLD and TOLD in 1 lesion (3%); TOLD and DWI in 1 lesion (3%); DCE-MRI only in 2 lesions (5%)].

**Table 2:** Central tendency (mean and median) and heterogeneity (SD, kurtosis, skewness) MRI parameters in 39 HCC lesions and liver parenchyma in 32 patients.

	Mean			Median			SD			Kurtosis			Skewness		
	**Liver**	**HCC**	***P*** *	**Liver**	**HCC**	***P*** *	**Liver**	**HCC**	***P*** *	**Liver**	**HCC**	***P*** *	**Liver**	**HCC**	***P*** *
F_a_	49 ± 45	247 ± 240	**<0.001**	47 ± 45	241 ± 250	**<0.001**	16.3 ± 13.0	83.0 ± 66.7	**<0.001**	4.3 ± 1.8	5.5 ± 6.0	0.754	0.76 ± 0.66	0.44 ± 1.34	0.232
F_p_	145 ± 123	112 ± 156	**0.048**	146 ± 126	103 ± 166	**0.010**	39.9 ± 32.5	78.6 ± 78.6	**0.020**	6.3 ± 14.0	64.1 ± 183.6	**0.005**	0.54 ± 1.57	4.00 ± 6.41	**<0.001**
F_t_	193 ± 122	358 ± 337	**0.023**	193 ± 125	342 ± 350	0.092	43.7 ± 36.2	144.3 ± 126.3	**<0.001**	4.5 ± 3.1	17.1 ± 54.9	0.969	0.62 ± 0.76	1.14 ± 3.11	0.984
ART	33.9 ± 27.9	78.9 ± 21.5	**<0.001**	33.0 ± 29.1	80.3 ± 24.2	**<0.001**	9.7 ± 5.1	13.1 ± 9.0	0.055	7.6 ± 10.5	30.1 ± 61.8	0.256	0.68 ± 1.71	−2.49 ± 4.37	**<0.001**
MTT	20.3 ± 12.3	21.2 ± 15.8	0.724	19.7 ± 12.1	18.8 ± 16.3	0.667	4.2 ± 2.9	10.9 ± 7.3	**<0.001**	5.2 ± 4.8	9.1 ± 17.4	0.938	0.90 ± 0.87	1.54 ± 2.06	0.337
DV	37.4 ± 22.7	34.6 ± 24.7	0.695	36.8 ± 22.8	34.0 ± 26.0	0.710	4.9 ± 3.4	9.9 ± 5.7	**<0.001**	3.7 ± 1.7	5.5 ± 10.8	0.240	0.58 ± 0.61	0.70 ± 1.59	0.468
R_2_* pre O_2_	55.3 ± 34.4	34.5 ± 15.1	**<0.001**	54.7 ± 34.8	33.1 ± 15.0	**<0.001**	7.6 ± 7.5	7.6 ± 5.2	0.681	4.0 ± 2.9	5.2 ± 4.3	0.433	0.35 ± 0.89	0.92 ± 1.09	0.055
R_2_* post O_2_	51.8 ± 30.8	33.7 ± 14.5	**<0.001**	51.5 ± 31.4	32.7 ± 14.6	**<0.001**	8.3 ± 10.7	8.0 ± 4.7	0.347	3.5 ± 1.8	4.0 ± 2.7	0.984	0.15 ± 0.64	0.72 ± 0.81	**0.011**
ΔR_2_*	−3.47 ± 10.0	−0.78 ± 8.74	0.281	−3.12 ± 8.8	−0.86 ± 7.48	0.264	8.8 ± 9.5	8.6 ± 5.2	0.638	3.9 ± 2.0	4.7 ± ± 3.6	0.830	−0.18 ± 0.72	−0.16 ± 0.94	0.799
R_1_ pre O_2_	1.76 ± 0.47	1.67 ± 0.94	0.052	1.76 ± 0.46	1.58 ± 0.73	**0.033**	0.12 ± 0.14	0.75 ± 3.31	**0.008**	3.7 ± 2.2	8.1 ± 17.7	0.531	−0.12 ± 0.76	0.72 ± 2.07	**0.012**
R_1_ post O_2_	1.87 ± 0.73	1.90 ± 1.79	**0.027**	1.77 ± 0.63	1.54 ± 0.61	**0.026**	2.26 ± 7.73	1.44 ± 5.48	0.422	71.4 ± 257.6	16.5 ± 34.5	0.327	2.13 ± 8.07	1.41 ± 2.76	0.153
ΔR_1_	0.11 ± 0.45	0.23 ± 1.15	0.176	0.01 ± 0.24	−0.05 ± 0.25	0.829	2.26 ± 7.73	1.06 ± 3.46	0.196	72.2 ± 257.4	17.6 ± 44.6	0.814	2.14 ± 8.08	1.50 ± 3.03	0.112
ADC	1.31 ± 0.58	1.43 ± 0.68	**<0.001**	1.31 ± 0.58	1.41 ± 0.70	**<0.001**	0.2 ± 0.1	0.3 ± 0.2	0.142	3.8 ± 2.2	4.0 ± 6.4	**0.030**	0.10 ± 0.79	0.33 ± 0.90	0.337

*P-values from Wilcoxon signed rank tests. Significant *P*-values are bolded. ADC = apparent diffusion coefficient (10^−3^ mm^2^/s), ART = arterial fraction (%), DV = distribution volume (%), F_a_ = arterial flow (ml/100 g/min), F_p_ = portal flow (ml/100 g/min), F_t_ = total flow (ml/100 g/min), MTT = mean transit time (s), R_1_ = longitudinal relaxation rate (s^−1^), R_2_* = transverse relaxation rate (s^−1^).

### Inter-tumour heterogeneity

**Figure 3:**
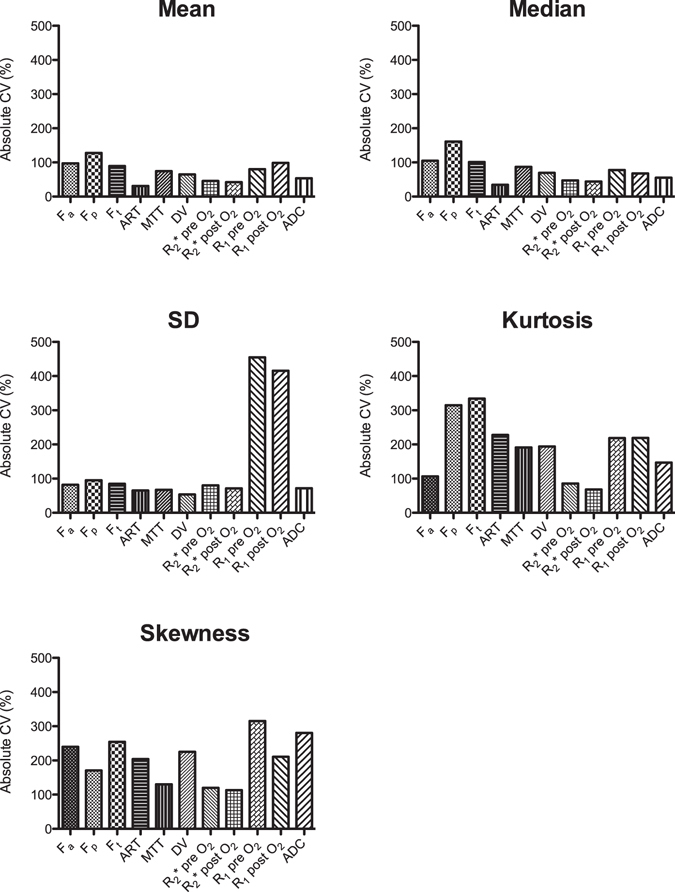
Inter-tumour variability in MRI parameters. Absolute coefficients of variation (CV) of mean, median, SD, kurtosis and skewness parameter values across 39 HCC tumours (in 32 patients). Kurtosis and skewness showed a substantially higher inter-tumour variability compared to mean and median values. ADC = apparent diffusion coefficient, ART = arterial fraction, DV = distribution volume, F_a_ = arterial flow, F_p_ = portal flow, F_t_ = total flow, MTT = mean transit time, R_1_ = longitudinal relaxation rate, R_2_* = transverse relaxation rate.

CV values of MRI parameters across tumours are shown in Fig. 3. CV values were markedly higher for kurtosis and skewness vs. mean and median values, indicative of higher inter-tumour variation for heterogeneity parameters. SD overall showed similar CV values as the mean and median values, except for a substantial higher CV observed for SD of R_1_ pre and post O_2_.

### Correlation between MRI methods

**Figure 4:**
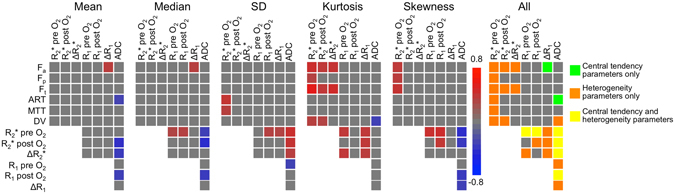
Heatmaps of correlations between mean, median, SD, kurtosis and skewness of MRI parameters in 39 HCC lesions (in 32 patients). Significant correlations (*P* < 0.05) are coloured according to the scale bar. The correlation between R_1_ pre and post O_2_ and DCE-MRI parameters was not assessed, because the baseline R_1_ measurements were used as input for the DCE-MRI modelling. A combined heatmap of all significant correlations between MRI features is shown on the right, illustrating additional information provided by heterogeneity parameters (SD, kurtosis and skewness). Significant correlations between DCE-MRI and BOLD were for example only seen for heterogeneity parameters and not for central tendency parameters (mean and median). ADC = apparent diffusion coefficient, ART = arterial fraction, DV = distribution volume, F_a_ = arterial flow, F_p_ = portal flow, F_t_ = total flow, MTT = mean transit time, R_1_ = longitudinal relaxation rate, R_2_* = transverse relaxation rate.

Figure 4 shows heatmaps of significant correlations found between the MRI parameter values in tumours. Numerical values of the correlations are listed in Supplementary Table S1. For the central tendency parameters, significant correlations were found between ADC values and parameters from all other sequences (DCE-MRI, BOLD and TOLD), between DCE-MRI and ΔR_1_ and between BOLD and TOLD values. However, no significant correlations were found between BOLD and DCE-MRI central tendency parameter values; while these parameters showed significant correlation with respect to their MRI heterogeneity features, which indicates that these parameters exhibited similar intra-tumour distribution, as illustrated in Fig. 2. Differences in correlations between mean and median MRI parameters at both field strengths were not significant (*P*-value range mean 0.119–0.949, median 0.098–0.981).

### Correlation with histopathology

**Figure 5:**
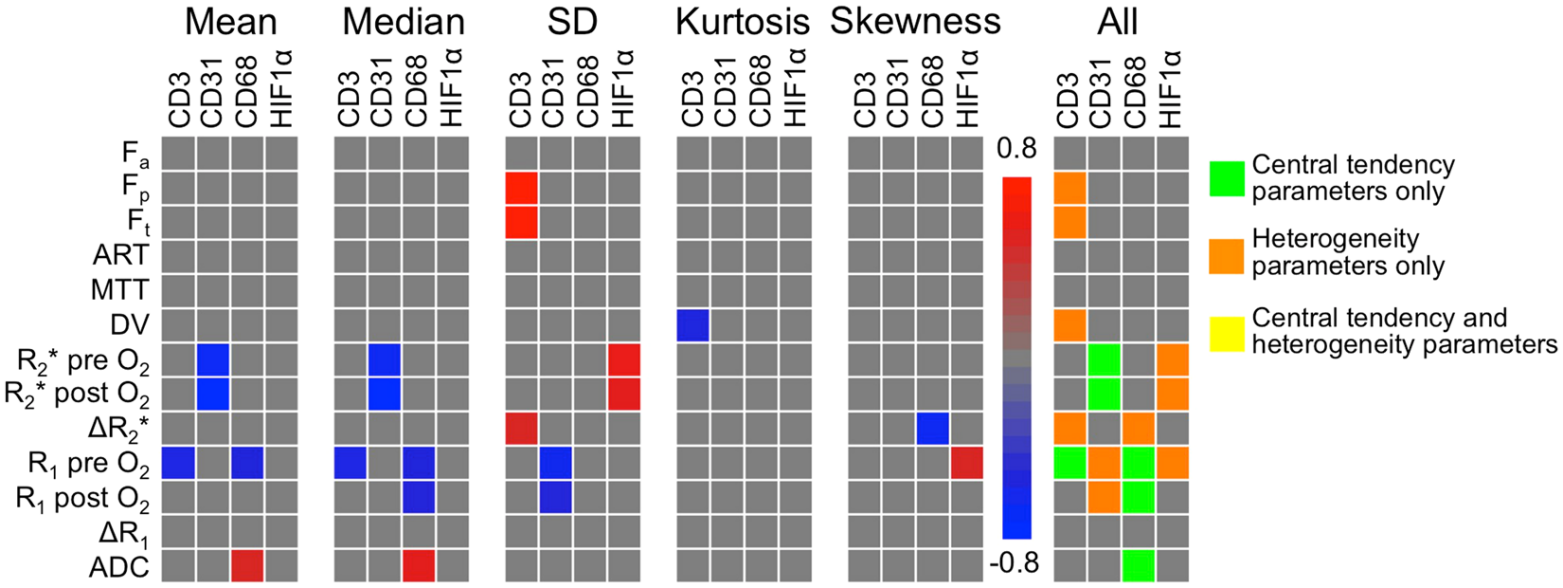
Heatmaps of correlations between MRI parameters and histopathology in 14 HCC lesions in 14 patients. Significant correlations (*P* < 0.05) are coloured according to the scale bar. A combined heatmap of all significant correlations between MRI features and histopathology is shown on the right, illustrating additional information provided by heterogeneity parameters (SD, kurtosis and skewness). For example, HIF1α tumour fractions correlated with heterogeneity parameters, but not with central tendency parameters (mean and median). ADC = apparent diffusion coefficient, ART = arterial fraction, CD3 = cluster of differentiation 3, CD31 = cluster of differentiation 31, CD68 = cluster of differentiation 68, DV = distribution volume, F_a_ = arterial flow, F_p_ = portal flow, F_t_ = total flow, HIF1α = hypoxia-inducible factor 1-alpha, MTT = mean transit time, R_1_ = longitudinal relaxation rate, R_2_* = transverse relaxation rate.

None of the MRI parameters showed a significant difference between histological grades (Kruskal-Wallis *P*-value range 0.085–0.410). Significant correlations between MRI parameters and histologically stained tumour fractions in 14 lesions (14 patients) are displayed in heatmaps in Fig. 5 (numerical values in Supplementary Table S2). CD3, CD31 and CD68 tumour fractions correlated moderately to strongly with both central tendency and heterogeneity parameter values. With regards of central tendency parameters, CD3 stained tumour fractions showed moderate significant negative correlations with mean and median R_1_ pre O_2_ (*P* < 0.029)_._ CD31 staining significantly negatively correlated with mean and median R_2_* pre and post O_2_ (*P* < 0.009). CD68 stained tumour fractions correlated negatively with mean and median R_1_ pre O_2_ and median R_1_ post O_2_ and positively with mean and median ADC (*P* < 0.050). HIF1α tumour fractions did not correlate with any of the central tendency parameters, but did show significant positive correlation with SD of R_2_* pre and post O_2_ and skewness of R_1_ pre O_2_ (*P* < 0.048).

### Gene expression analysis

**Figure 6:**
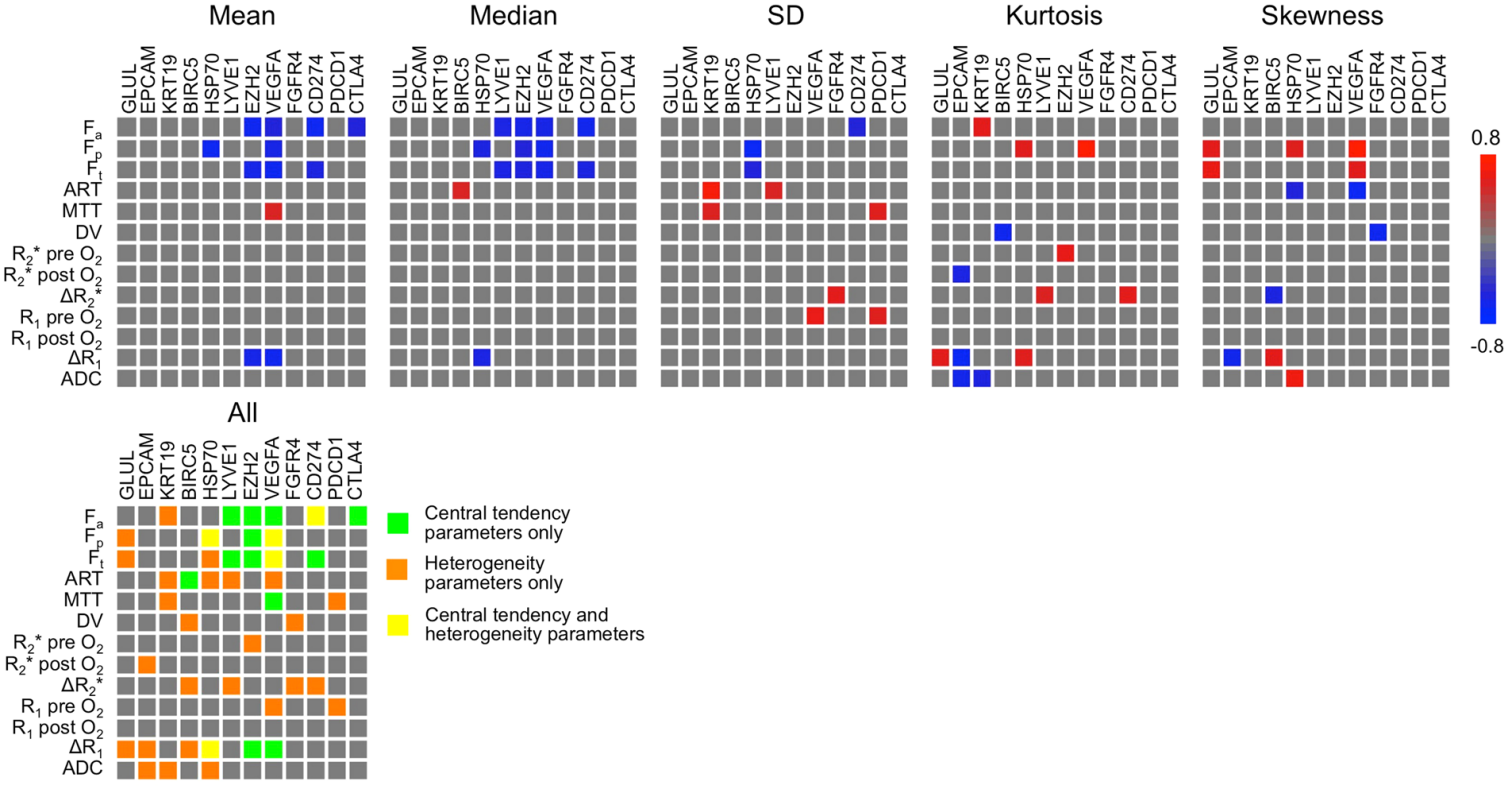
Heatmaps of correlations between MRI parameters and gene expression levels in 14 HCC lesions in 14 patients. Significant correlations (*P* < 0.05) are coloured according to the scale bar. A combined heatmap of all MRI features is shown at the figure bottom, illustrating additional information provided by heterogeneity parameters (SD, kurtosis and skewness) compared to central tendency parameters (mean and median) with respect to correlations between MRI and gene expression levels. ADC = apparent diffusion coefficient, ART = arterial fraction, *BIRC5* = Baculoviral IAP repeat containing 5, *CD274* = cluster of differentiation 274, *CTLA4* = cytotoxic T-lymphocyte-associated protein 4, DV = distribution volume, *EPCAM* = epithelial cell adhesion molecule, *EZH2* = enhancer of zeste homolog 2, F_a_ = arterial flow, *FGFR4* = fibroblast growth factor receptor 4, F_p_ = portal flow, F_t_ = total flow, *GLUL* = glutamate-ammonia ligase, *HSP70* = 70 kilodalton heat shock protein, *KRT19* = keratin 19; *LYVE1* = lymphatic vessel endothelial hyaluronan receptor 1, MTT = mean transit time, R_1_ = longitudinal relaxation rate, R_2_* = transverse relaxation rate, *VEGFA* = vascular endothelial growth factor A.

The distribution of HCC molecular subclasses was as follows: S1 (n = 6, 43%), S2 (n = 3, 21%), S3 (n = 5, 36%). No significant differences between MRI parameters in the different subclasses were observed (*P*-value range 0.160–0.970). MRI central tendency and heterogeneity features were complementary in terms of correlations with gene expression levels, as illustrated in Fig. 6 (numerical correlation values in Supplementary Table S3). The expression level of *CTLA4* only correlated with central tendency parameters, while expression of *GLUL*, *FGFR4*, tumour stemness markers *KRT19* and *EPCAM* and immune checkpoint *PDCD1* showed significant correlations with MRI heterogeneity parameters only. The expression levels of early HCC markers *BIRC5, HSP70*, *LYVE* and *EZH2*, angiogenesis marker *VEGFA* and immune checkpoint *CD274* significantly correlated with both central tendency and heterogeneity parameters.

## Discussion

In this study we quantified intra- and inter-tumour heterogeneity of HCC lesions using quantitative mpMRI and correlated the mpMRI parameters with histopathological and gene expression analysis in a subset of patients.

Intra-tumour heterogeneity, assessed by the presence of leptokurtosis in the MRI parameters, was observed in all lesions. DCE-MRI and TOLD parameters showed a significantly higher kurtosis compared to ADC, which suggests that intra-tumour heterogeneity in HCC is mainly caused by spatial variation in presence of (functional) vasculature/oxygenation rather than intra-tumoural differences in cellularity.

A higher inter-tumour variation was found for the heterogeneity parameters compared to the mean and median parameter values. This finding suggests that the proposed heterogeneity analysis could potentially allow for a more extensive differentiation between tumours than using the more traditional analysis of central tendency parameter values in ROIs.

With respect to inter-sequence correlations, we found that DCE-MRI and BOLD MRI central tendency parameters did not correlate, while heterogeneity features of these two sequences showed a significant correlation, indicating that DCE-MRI and BOLD-MRI parameters exhibited a similar intra-tumour distribution. Baudelet *et al*. found that DCE-MRI and BOLD MRI were also largely complementary for the characterization of vasculature in a preclinical fibrosarcoma model^43^. A possible explanation for the lack of a clear correlation between the techniques could be due to the fact that BOLD-MRI has intrinsic low sensitivity in tumour blood vessels with low haematocrit, as opposed to DCE-MRI, in which contrast agent influx is not affected by haematocrit^43^.

Significant correlations were found between BOLD, TOLD and DWI central tendency parameters and histopathology. The negative correlations between R_1_ pre O_2_ and CD3- and CD68-stained tumour fractions and the positive correlation between ADC and CD68 tumour fractions may be explained by a possible increase in interstitial fluid associated with inflammation^44^. The negative correlation between R_2_* (pre and post O_2_) and CD31 may be due to the fact that tumours with higher vascularity will most probably be less hypoxic and therefore exhibit a lower R_2_*.

Strong significant correlations were observed between gene expression levels and several MRI parameters. Poor tumour perfusion (low flow/ART and high MTT) was associated with high expression of HCC markers, *VEGFA* and immune checkpoints *CD274* and *CTLA4*. The relation with HCC markers may be related to tumour progression, which is associated with decreased arterial flow, secondary to increased interstitial pressure and resulting closure of arterial capillaries^45^, and higher expression of HCC markers, including *HSP70* and *LYVE1* ^46, 47^. The negative correlation between perfusion and *VEGFA* expression could potentially be explained by the fact that decreased perfusion may be associated with hypoxia, which triggers expression of *VEGFA* ^48^. However, counterintuitively, *VEGFA* expression was not associated with mean or median BOLD parameters and negatively associated with ΔR_1_, which suggests absence of correlation between MRI-measured hypoxia and expression of *VEGFA*. The underlying physiology of the negative correlations between tumour perfusion and *CTLA4* and *CD274* expression is unclear and needs to be verified in a larger study. The expression of immune checkpoints has shown to be positively associated with tumour progression in lung cancer^49^, but no data has been reported on this association in HCC. Interestingly, significant correlations with expression of several genes, including stemness markers and immunotherapy target *PDCD1*, were only observed with heterogeneity parameters, which further illustrates the complementary properties of central tendency and heterogeneity parameters in terms of tumour characterization.

In general, limited data is available on radiogenomics in HCC. There are a few reports that compare CT imaging traits with HCC genomics analysis^10, 11^. However, these studies have assessed only qualitative metrics, which are limited by inter- and intra-observer variability. The quantitative assessment of heterogeneity histogram features of functional mpMRI in the current study is probably less prone to inter-observer variability. Nevertheless, the proposed heterogeneity features can potentially be confounded by image noise, which introduces non-biological heterogeneity, and by inaccuracies in parameter estimation of single pixels^50^. However, we found that the HCC lesions exhibited more intra-tissue heterogeneity than liver parenchyma, which indicates that the observed intra-tumour heterogeneity in the HCC lesions is likely biological in nature.

There are a few published reports on the use of imaging to predict and assess immunotherapy outcome^51^. Jajamovich *et al*. found a significant correlation between ADC and a gene signature related to dendritic cell maturation in glioblastoma, which can be used to stratify patients with immunogenic tumours for immunotherapy^52^. Mayerhoefer *et al*. found that DWI is a promising technique for the evaluation of immunotherapy response in lymphoma patients^53^. The potential value of DWI for immunotherapy was also reported by Qin *et al*., who found that the decrease or stabilization of the lesion volume on ADC maps, after an initial increase after immunotherapy, was predictive of therapeutic benefit in glioblastoma^54^. Interestingly, we did not find significant correlations between ADC and any of the immunotherapy targets. The role of DWI for the prediction of immunotherapy outcome in HCC needs to be established in future studies. There is no published data assessing HCC response to immunotherapy, given the recent introduction of this therapy in HCC. More studies are needed to determine the exact value of mpMRI for the prediction of immunotherapy efficacy in HCC and other cancer types.

While mpMRI proved promising for the non-invasive assessment of histopathological and genomics properties of HCC lesions in our study, the separate imaging, histopathological and genomics measurements also provided independent information on tumour properties. For example, none of the mpMRI features could distinguish between different histopathological grades. We therefore do not believe that mpMRI could fully replace histopathologic assessment in HCC. The mpMRI analysis could rather be used to improve the characterization of HCC lesions in biopsied or resected samples, by providing mutual and additional information on tumour properties on a whole-tumour level^12^. Nevertheless, in the majority of patients HCC is diagnosed based on imaging alone, without histologic confirmation^55^. Treatment stratification in those patients without biopsy could ultimately benefit from knowledge of the correlation of imaging parameters with histopathological and genomics properties of HCC lesions in a training set of resected HCC samples.

The findings described in our study may have substantial clinical implications for characterization and treatment stratification of HCC. Tumours with high intra-tumour heterogeneity generally have a poorer prognosis, which may be secondary to a more aggressive biology or treatment resistance^56^. The observed correlations between MRI features and the expression of potential therapeutic markers for targeted molecular or immunotherapy of HCC^57^ suggest that the proposed mpMRI histogram analysis could be used to noninvasively predict treatment outcome at baseline. Knowledge of the expression of therapeutic targets using non-invasive imaging could aid in the stratification of patient-tailored, personalized treatment. A future study should assess the proposed mpMRI heterogeneity analysis as a predictor of response to immunotherapy. In such study, it could also be assessed which (combination of) MRI features have the highest diagnostic performance for prediction of treatment response.

Our study has several limitations. Assessment of intra-tumour heterogeneity was not feasible for the histopathology and genomics analysis, because only a small part of the lesion was available for these analyses. Another limitation was the relatively small sample size of HCC lesions for histopathology and genomics. Moreover, there was variability between MRI field strengths on which the patients were scanned, which could affect MRI parameter estimation. Nevertheless, the correlations between MRI parameters were not significantly different between both field strengths. Finally, the correlation of the mpMRI features with clinical parameters such as survival and treatment response was not assessed in this initial study.

In conclusion, our results show that central tendency and heterogeneity features of mpMRI data of HCC tumours are complementary in terms of correlations between sequences, with histopathology and with gene expression levels. The proposed histogram analysis is therefore promising for non-invasive HCC characterization on the imaging, immunohistochemical and genomics level.

## Electronic supplementary material

Supplementary Information
