# Supplementary material for: Quantification of hepatocellular carcinoma heterogeneity with multiparametric magnetic resonance imaging
Source: Sci Rep. 2017 May 26;7:2452. doi: 10.1038/s41598-017-02706-z (PMC5446396; doi:10.1038/s41598-017-02706-z)
Supplement: Supplementary file 1 — Supplementary Information [file 41598_2017_2706_MOESM1_ESM.pdf]

Supplementary Information

Quantification of hepatocellular carcinoma heterogeneity with multiparametric magnetic resonance imaging.

Stefanie J Hectors[1,2], Mathilde Wagner[1,2,3], Octavia Bane[1,2], Cecilia Besa[1,2], Sara Lewis[2], Romain Remark[4], Nelson Chen[1,2], M. Isabel Fiel[5], Hongfa Zhu[5], Sacha Gnjatic[6], Miriam Merad[4], Yujin Hoshida[7], Bachir Taouli[1,2]

[1] Translational and Molecular Imaging Institute, Icahn School of Medicine at Mount Sinai, New York, NY, United States

[2] Department of Radiology, Icahn School of Medicine at Mount Sinai, New York, NY, United States

[3] Sorbonne Universités, UPMC, Department of Radiology, Hôpital Pitié-Salpêtrière, Assistance Publique-Hôpitaux de Paris, Paris, France

[4] Immunology Institute, Icahn School of Medicine at Mount Sinai, New York, NY, United States

[5] Department of Pathology, Icahn School of Medicine at Mount Sinai, New York, NY, United States

[6] Oncological Science, Icahn School of Medicine at Mount Sinai, New York, NY, United States

[7] Department of Medicine/Division of Liver Diseases, Icahn School of Medicine at Mount Sinai, New York, NY, United States

Table S1: Significant correlations between mean, median, SD, kurtosis and skewness of parameters in ROIs in 39 HCC lesions (32 patients).

**Mean**		**Median**		**SD**		**Kurtosis**		**Skewness**	
F_a – ΔR_1	r=0.365, *P*=0.012	F_a – ΔR_1	r=0.349 *P*=0.030	ART – R_2* pre O_2	r=0.418, *P*=0.009	F_a – R_2* pre O_2	r=0.461, *P*=0.003	F_a – R_2* pre O_2	r=0.383, *P*=0.017
ART – ADC	r=-0.322, *P*=0.046	R_2* pre O_2 – R_1 pre O_2	r=0.318, *P*=0.049	MTT – R_2* pre O_2	r=0.347, *P*=0.031	F_a – R_2* post O_2	r=0.390, *P*=0.015	F_p – R_2* pre O_2	r=0.370, *P*=0.021
R_2* post – ADC	r=-0.440, *P*=0.005	R_2* pre O_2 – R_1 post O_2	r=0.334, *P*=0.038	MTT – R_1 pre O_2	r=0.389, *P*=0.015	F_a – ΔR_2*	r=0.429, *P*=0.007	F_t – R_2* pre O_2	r=0.424, *P*=0.008
ΔR_2* – ADC	r=-0.383, *P*=0.017	R_2* pre O_2 – ADC	r=-0.350, *P*=0.030	DV – R_1 pre O_2	r=0.458, *P*=0.004	F_p – R_2* pre O_2	r=0.334, *P*=0.038	R_2* pre O_2 – R_1 pre O_2	r=0.451, *P*=0.004
R_1 post O_2 – ADC	r=-0.348, *P*=0.031	R_2* post O_2 – ADC	r=-0.477, *P*=0.002	R_2* pre O_2 – R_1 post O_2	r=0.338, *P*=0.036	F_p – R_1 pre O_2	r=0.330, *P*=0.041	R_2* pre O_2 – R_1 post O_2	r=0.473, *P*=0.003
		R_1 post O_2 – ADC	r=-0.388, *P*=0.015	R_2* pre O_2 – ΔR_1	r=0.326, *P*=0.044	F_t – R_2* pre O_2	r=0.590, P<0.001	R_2* pre O_2 – ADC	r=-0.461, *P*=0.003
				R_2* pre O_2 – ADC	r=0.457, *P*=0.004	F_t – R_2* post O_2	r=0.397, *P*=0.013	R_2* post O_2 – R_1 post O_2	r=0.404, *P*=0.011
				R_2* post O_2 – ADC	r=0.425, *P*=0.007	F_t – ΔR_2*	r=0.381, *P*=0.002	R_2* post O_2 – ADC	r=-0.390, *P*=0.015
				ΔR_2* – ADC	r=0.351, *P*=0.029	DV – R_2* pre O_2	r=0.350, *P*=0.030	R_1 post O_2 – ADC	r=-0.383, *P*=0.017
				R_1 pre O_2 – ADC	r=-0.334, *P*=0.038	DV – R_2* post O_2	r=0.325, *P*=0.044	ΔR_1 – ADC	r=-0.349, *P*=0.030
						DV – ADC	r=-0.340, *P*=0.035		
						R_2* pre O_2 – R_1 pre O_2	r=0.391, *P*=0.014		
						R_2* pre O_2 – ΔR_1	r=0.396, *P*=0.013		
						R_2* post O_2 – ΔR_1	r=0.394, *P*=0.014		
						ΔR_2* – R_1 pre O_2	r=0.398, *P*=0.013		
						ΔR_2* – ΔR_1	r=0.375, *P*=0.019		

ADC = apparent diffusion coefficient, ART = arterial fraction, DV = distribution volume, F_a = arterial flow, F_p = portal flow, F_t = total flow, MTT = mean transit time, R_1 = longitudinal relaxation rate, R_2* = transverse relaxation rate

Table S2: Significant correlations of MRI parameters with histopathology in 14 HCC lesions (14 patients).

**Mean**		**Median**		**SD**		**Kurtosis**		**Skewness**	
CD3 – R_1 pre O_2	r=-0.591, P=0.029	CD3 – R_1 pre O_2	r=-0.591, P=0.029	CD3 – F_p	r=0.771, P=0.002	CD3 – DV	r=-0.565, P=0.038	CD68 – ΔR_2^*	r=-0.684, P=0.009
CD31 – R_2^* pre O_2	r=-0.701, P=0.007	CD31 – R_2^* pre O_2	r=-0.684, P=0.009	CD3 – F_t	r=0.749, P=0.003			HIF1α – R_1 pre O_2	r=0.543, P=0.048
CD31 – R_2^* post O_2	r=-0.851, P=0.000	CD31 – R_2^* post O_2	r=-0.864, P=0.000	CD3 – ΔR_2^*	r=0.538, P=0.050				
CD68 – R_1 pre O_2	r=-0.560, P=0.040	CD68 – R_1 pre O_2	r=-0.560, P=0.040	CD31 – R_1 pre O_2	r=-0.666, P=0.011				
CD68 – ADC	r=0.538, P=0.050	CD68 – R_1 post O_2	r=-0.552, P=0.044	CD31 – R_1 post O_2	r=-0.538, P=0.050				
		CD68 – ADC	r=0.591, P=0.029	HIF1α - R_2^* pre O_2	r=0.622, P=0.020				
				HIF1α - R_2^* post O_2	r=0.596, P=0.028				

ADC = apparent diffusion coefficient, ART = arterial fraction, CD3 = cluster of differentiation 3, CD31 = cluster of differentiation 31, CD68 = cluster of differentiation 68, DV = distribution volume, F_a = arterial flow, F_p = portal flow, F_t = total flow, HIF1α = hypoxia-inducible factor 1-alpha, MTT = mean transit time, R_1 = longitudinal relaxation rate, R_2^* = transverse relaxation rate

Table S3: Significant correlations of MRI parameters with gene expression levels in 14 HCC lesions (14 patients).

**Mean**		**Median**		**SD**		**Kurtosis**		**Skewness**	
HSP70 – F_p	r=-0.675, *P*=0.010	BIRC5 – ART	r=0.536, *P*=0.048	KRT19 – ART	r=0.697, *P*=0.007	GLUL – ΔR_1	r=0.587, *P*=0.030	GLUL – F_p	r=0.596, *P*=0.028
EZH2 – F_a	r=-0.670, *P*=0.011	HSP70 – F_p	r=-0.578, *P*=0.030	KRT19 – MTT	r=0.547, *P*=0.046	EPCAM – R_2* post O_2	r=-0.591, *P*=0.029	GLUL – F_t	r=0.631, *P*=0.018
EZH2 – F_t	r=-0.622, *P*=0.020	HSP70 – ΔR_1	r=-0.591, *P*=0.029	HSP70 – F_p	r=-0.697, *P*=0.007	EPCAM – ΔR_1	r=-0.692, *P*=0.008	EPCAM – ΔR_1	r=-0.644, *P*=0.015
EZH2 – ΔR_1	r=-0.622, *P*=0.020	LYVE1 – F_a	r=-0.591, *P*=0.029	HSP70 – Ft	r=-0.547, *P*=0.046	EPCAM – ADC	r=-0.635, *P*=0.017	BIRC5 – ΔR_2*	r=-0.582, *P*=0.032
VEGFA – F_a	r=-0.547, *P*=0.046	LYVE1 – F_t	r=-0.596, *P*=0.028	LYVE1 – ART	r=0.538, *P*=0.050	KRT19 – F_a	r=0.596, *P*=0.028	BIRC5 – ΔR_1	r=0.600, *P*=0.026
VEGFA – F_p	r=-0.609, *P*=0.024	EZH2 – F_a	r=-0.622, *P*=0.020	VEGFA – R_1 pre O_2	r=0.622, *P*=0.020	KRT19 – ADC	r=-0.574, *P*=0.035	HSP70 – F_p	r=0.569, *P*=0.037
VEGFA – F_t	r=-0.666, *P*=0.011	EZH2 – F_p	r=-0.538, *P*=0.047	FGFR4 – ΔR_2*	r=0.596, *P*=0.028	BIRC5 – DV	r=-0.662, *P*=0.012	HSP70 – ART	r=-0.552, *P*=0.044
VEGFA – MTT	r=0.538, *P*=0.050	EZH2 – F_t	r=-0.565, *P*=0.038	CD274 – F_a	r=-0.543, *P*=0.048	HSP70 – F_p	r=0.582, *P*=0.032	HSP70 – ADC	r=0.622, *P*=0.012
VEGFA – ΔR_1	r=-0.662, *P*=0.012	VEGFA – F_a	r=-0.609, *P*=0.024	PDCD1 – MTT	r=0.565, *P*=0.038	HSP70 – ΔR_1	r=0.574, *P*=0.035	VEGFA – F_p	r=0.754, *P*=0.003
CD274 – F_a	r=-0.675, *P*=0.010	VEGFA – F_p	r=-0.653, *P*=0.011	PDCD1 – R_1 pre O_2	r=0.569, *P*=0.037	LYVE1 – ΔR_2*	r=0.547, *P*=0.046	VEGFA – F_t	r=0.578, *P*=0.033
CD274 – F_t	r=-0.574, *P*=0.035	VEGFA – F_t	r=-0.622, *P*=0.020			EZH2 – R_2* pre O_2	r=0.618, *P*=0.021	VEGFA – ART	r=-0.684, *P*=0.009
CTLA4 – F_a	r=-0.552, *P*=0.044	CD274 – F_a	r=-0.653, *P*=0.014			VEGFA – F_p	r=0.749, *P*=0.003	FGFR4 – DV	r=-0.670, *P*=0.011
		CD274 – F_t	r=-0.578, *P*=0.033			CD274 – ΔR_2*	r=0.591, *P*=0.029		

ADC = apparent diffusion coefficient, ART = arterial fraction, BIRC5 = Baculoviral IAP repeat containing 5, CD274 = cluster of differentiation 274, CTLA4 = cytotoxic T-lymphocyte-associated protein 4, DV = distribution volume, EPCAM = epithelial cell adhesion molecule, EZH2 = enhancer of zeste homolog 2, F_a = arterial flow, FGFR4 = fibroblast growth factor receptor 4, F_p = portal flow, F_t = total flow, GLUL = glutamate-ammonia ligase, HSP70 = 70 kilodalton heat shock protein, KRT19 = keratin 19, LYVE1 = lymphatic vessel endothelial hyaluronan receptor 1, MTT = mean transit time, R_1 = longitudinal relaxation rate, R_2* = transverse relaxation rate, VEGFA = vascular endothelial growth factor A
